# The Diagnosis Accuracy of PLA2R-AB in the Diagnosis of Idiopathic Membranous Nephropathy: A Meta-Analysis

**DOI:** 10.1371/journal.pone.0104936

**Published:** 2014-08-19

**Authors:** Yu Du, Junhua Li, Fan He, Yongman Lv, Wei Liu, Ping Wu, Jiao Huang, Sheng Wei, Hongyu Gao

**Affiliations:** 1 Department of Nephrology, Tongji hospital affiliated to Tongji medical college, Huazhong University of Science and Technology, Wuhan, Hubei, China; 2 Department of Pathophysiology, Tongji Medical College, Huazhong University of Science and Technology, Wuhan, Hubei, China; 3 Department of Epidemiology and Biostatistics, School of Public Health, Tongji Medical College, Huazhong University of Science and Technology, Wuhan, Hubei, China; IRCSS - Istituto di Ricerche Farmacologiche Mario Negri, Italy

## Abstract

**Background:**

The presence of antibodies against the M-type phospholipase A2 receptor (PLA2R-AB) is considered to be a promising serological diagnostic biomarker of idiopathic membranous nephropathy (iMN). However, controversy remains about the diagnostic accuracy of serum PLA2R-AB testing. Here, we performed a comprehensive meta-analysis to assess the overall diagnostic value of serum PLA2R-AB testing in iMN detection.

**Methods:**

PubMed, Embase, and CNKI (Chinese National Knowledge Infrastructure) were searched for relevant original articles through January 31, 2014. The summary sensitivity, specificity, positive likelihood ratio, negative likelihood ratio and diagnostic odds ratio (DOR) were estimated using the bivariate model. The heterogeneity among studies was explored by subgroup and meta-regression analysis.

**Results:**

9 articles, including 15 studies, were eventually identified with a total of 2212 patients. The summary sensitivity of all studies is 78% (95% CI: 66% to 87%) and the specificity is 99% (95% CI: 96% to 100%). The summary positive and negative likelihood ratios are 96.1 (95% CI, 19.5 to 472.1) and 0.22 (95% CI: 0.14 to 0.35), respectively. The DOR is 437 (95%CI, 74 to 2592). The subgroup analysis and meta-regression suggest the test interval is the main source of heterogeneity.

**Conclusions:**

Serum PLA2R-AB testing is a useful tool to detect iMN. In addition, considering the high heterogeneity and potential publication bias, further high quality studies are needed in the future.

## Introduction

Membranous nephropathy (MN) is one of the leading causes of nephritic syndrome in adults [1]. The disease is characterized by the formation of subepithelial immune deposits and complement mediated proteinuria [2], [3]. Approximately 80% of all cases are referred to as ‘idiopathic’ MN (iMN) because they have no known etiology. The remaining 20–25% cases of MN are classified as ‘secondary’ cases due to their association with co-morbid clinical conditions such as systemic lupus erythematodes (SLE), cancer, viral or bacterial infection, and/or drug intoxication [4], [5]. In order to substantially improve the management and clinical outcome of patients with MN, it is extremely important to ensure reliable differential diagnoses between idiopathic and secondary MN [2], [6].

The M-type phospholipase A2 receptor (PLA2R) was recently identified as a major target antigen in autoimmune idiopathic membranous nephropathy [7]. Several studies have indicated that about 70–80% of patients with iMN tested positive for circulating antibodies against PLA2R(PLA2R-AB). Conversely, patients with secondary MN or other proteinuric disease tested negative for PLA2R-AB [8]. Since the level of PLA2R-AB correlates with clinical disease activity, it could be used to monitor a patient's response to treatment. This suggests that serum PLA2R-AB may serve as promising alternative diagnostic biomarker for iMN [7], [9], [10].

Compared with histological examination, serological testing for circulating PLA2R-AB is both more convenient and safer than traditional pathological examination. While a renal biopsy is invasive and may cause glomerular injury or other more serious complications, testing serum PLA2R-AB provides a quick disease detection method for clinicians. However, a series of prior studies showed that serum PLA2R-AB diagnoses were conflicting and could be extremely varied. For example, the sensitivity of PLA2R-AB tests ranged from 52% to 98.4% across all current studies [11]–[15]. Although PLA2R-AB may be a new tool for iMN diagnosis, its efficacy still remains controversial. Therefore, to comprehensively assess the diagnostic value of serum PLA2R-AB testing for iMN, we undertook the present meta-analysis to assess the overall diagnostic sensitivity and specificity of PLA2R-AB testing in patients with idiopathic membranous nephropathy.

## Materials and Methods

### Search strategy and study selection

PubMed, Embase, and CNKI (Chinese National Knowledge Infrastructure) were searched to identify eligible studies published prior to January 1st, 2014. The search terms used were “phospholipase A2 receptor antibody”, “PLA2R AB” and “membranous nephropathy”. Studies were also identified by the references cited in selected articles and were then searched manually. Two reviewers (YD and JH) independently determined study eligibility and disagreement between reviewers was resolved by consensus.

### Selection criteria

Studies were included in the current meta-analysis if they met the following criteria: (1) evaluation of the accuracy of PLA2R-AB testing on iMN diagnosis; (2) estimation of the sensitivity and specificity of the PLA2R-AB test; and (3) using of biopsy test results as a gold standard. Cases were excluded from this study for the following reason(s) (1): were a case report, review, letter, editorial, or comment; (2) had not performed any tests on serum levels of the PLA2R antibody; or (3) did not provide sufficient data. If studies had overlapping subjects, only the study with the largest sample size was included in the final analysis. Finally, since immunosuppressive therapy could affect the serum levels of PLA2R-AB, patients who received immunosuppressive therapy were excluded from our meta-analysis.

### Data extraction and quality assessment of studies

Two reviewers independently reviewed the articles and extracted the following data from all eligible publications: first author, year of publication, total number of patients, race, mean age, proportion of females, test methods, sensitivity, specificity, funding source and methodological quality.

The methodological quality of studies was evaluated independently by two reviewers (YD and JH) with the quality assessment of diagnostic accuracy studies (QUADAS) tool [16]. Component analysis was performed by creating a proportional bar graph for each of the 14 individual criteria. Each item was scored ‘yes’ if reported, ‘no’ if not reported, or ‘unclear’ if there were insufficient data to make a definitive assessment.

### Data analysis

A random-effects model was used to calculate the average sensitivity, specificity, positive likelihood ratio, negative likelihood ratio, and diagnostic odds ratio (DOR) across studies. A summary receiver operator characteristic (SROC) curve was then used to plot the consistency of results among all studies as well as the accuracy of the test. Both the χ^2_^test and I^2^ were used to detect statistically significant heterogeneity. Subgroup analyses were performed to identify factors which may be sources of heterogeneity. Such factors included: race, type of controls (e.g. patients with other kidney disease versus healthy controls), sample size, PLA2R-AB testing methods (e.g. Western blotting versus indirect immunofluorescence), test time intervals (e.g. testing simultaneously with the biopsy test versus testing after the biopsy test), and funding sources (e.g. sponsorship from government or corporate monies). These factors were then included as covariates in a meta-regression analysis to determine if they were statistically significant sources of heterogeneity. Lastly, a sensitivity analysis was conducted to assess the influence of each study on the overall parameter estimates.

A funnel plot and the effective sample size regression test for asymmetry were used to explore potential publication biases [17]. All analyses were performed in *mdias* module in Stata 10.0 (College Station, TX, USA).

## Results

### Study characteristics

**Figure 1:**
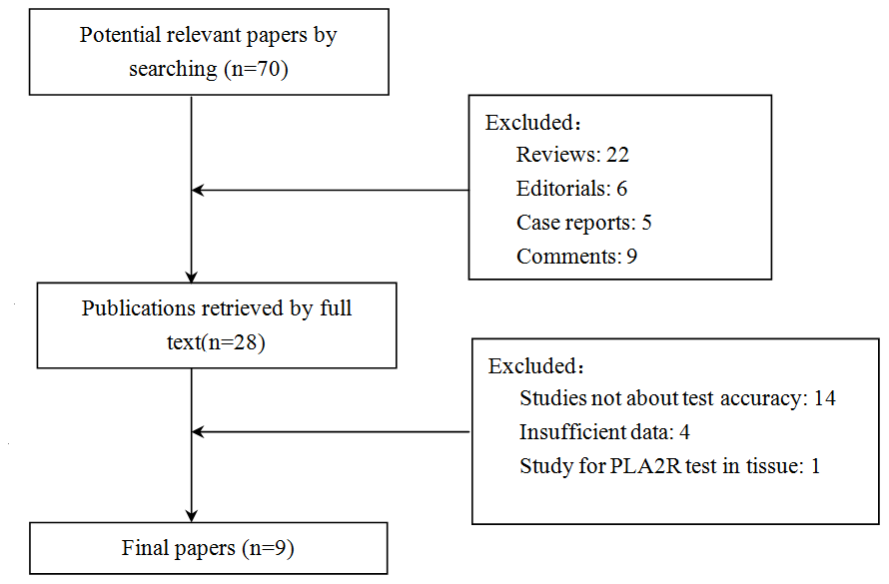
Flowchart for identification of studies.

As shown in Figure 1, there were 70 potentially relevant articles found in our search. Forty-two articles met exclusion criteria (22 reviews, 6 editorials, 5 case reports and 9 comments). The remaining 28 articles were retrieved for full-text review. Nineteen were excluded (14 studies did not investigate the test accuracy, 4 studies had insufficient data and 1 study only investigated PLA2R-AB in tissue). Finally, 9 articles, including 15 studies, were included in the present meta-analysis.

The characteristics of the included studies are shown in Table 1. The total population of the studies was 2212. Three articles used patients with other kidney disease as their patient controls. In total, this left 6 articles that had healthy patients and patients with other kidney disease as controls. There were 6 prospective studies and 9 retrospective studies. A more detailed description of the included studies is shown in Table S1.

**Table 1:** Characteristics of included eligible studies.

Year	Author	Country	Race	Case	Control1	Control2	TP no.	FP1* no.	FP2** no	FN, no.	TN1*, no.	TN2**.no	Se. %	Sp1* %	Sp2**%
2009	Beck	USA	Caucasian	12	30	30	9	0	0	3	30	30	75.0	100	100
2011	Qin	China	Asia	60	46	-	49	5	-	11	41	-	81.7	89.1	-
2011	Hoxha	Germany	Caucasian	43	107	153	21	0	0	22	107	153	48.8	100	100
2012	Murtas	Italian	Caucasian	186	92	96	111	0	0	75	92	96	59.7	100	100
2012	Hoxha	Germany	Caucasian	61	27	-	60	0	-	1	27	-	98.4	100	-
2013	Svobodova	Czech	Caucasian	28	3	-	18	2	-	10	1	-	64.3	33.3	-
2013	Dahnrich	West Europe	Caucasian	200	573	291	193	0	1	7	573	290	96.5	100	99.7
2013	Oh	Korea	Asia	100	9	14	69	2	0	31	7	14	69.0	77.8	100
2012	Zhou	China	Asia	20	26	5	15	2	0	5	24	5	75.0	92.3	100

*Other kidney disease control.

**Health control.

### Methodological quality of included studies

**Figure 2:**
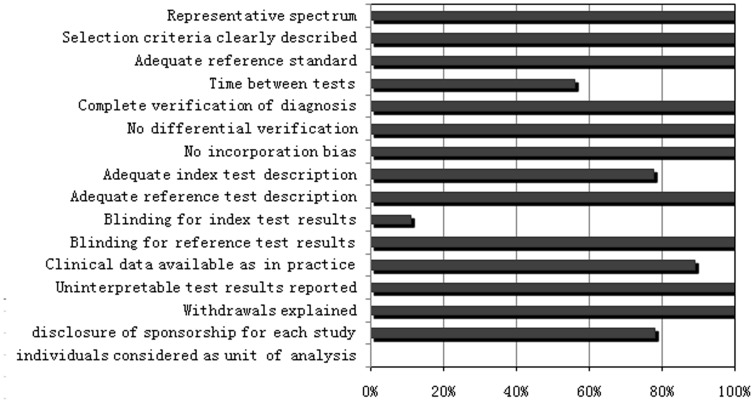
Proportion of all 14 quality assessment of diagnostic accuracy studies tool criteria that were fulfilled for the studies included in the meta-analysis.

The methodological quality assessment for included studies is shown in Figure 2. The overall quality of the eligible studies was not robust. Although almost all studies passed quality items 1, 2, 3, 5, 6, 7, 9, 11, 13 and 14, only one study reported blinding the reference PLA2R-AB test result to the results of the biopsy test (Item 10) [18]. Four studies had not reported whether or not the time period between reference standard and index test was short enough (Item4). Two studies had not reported the execution of the biopsy test insufficient detail (Item 8). One study did not clearly state clinical data, such as when the PLA2R-AB test results were interpreted (Item 12). Two studies (Dahnrich and Zhou) did not mention their respective sponsorship sources. The rest of the seven studies were supported predominantly by grants from government and foundation sources, but Beck's financial assistance came from a pharmaceutical corporation. It should be noted that since we did not obtain the individual data from every study, we did not conduct a meta-analysis that considered individuals as a unit of analysis.

### Meta-analysis

**Figure 3:**
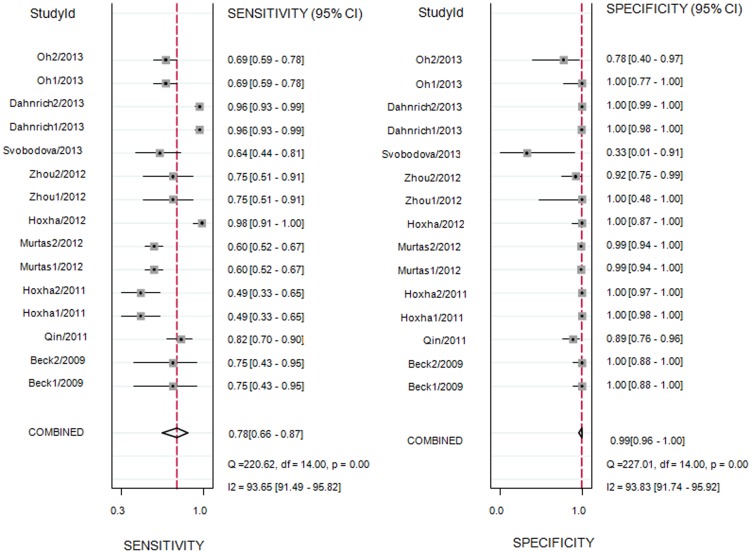
Forrest plots of the sensitivity and specificity of each individual study, summary sensitivity and specificity and I^2^ statistic for heterogeneity.

**Figure 4:**
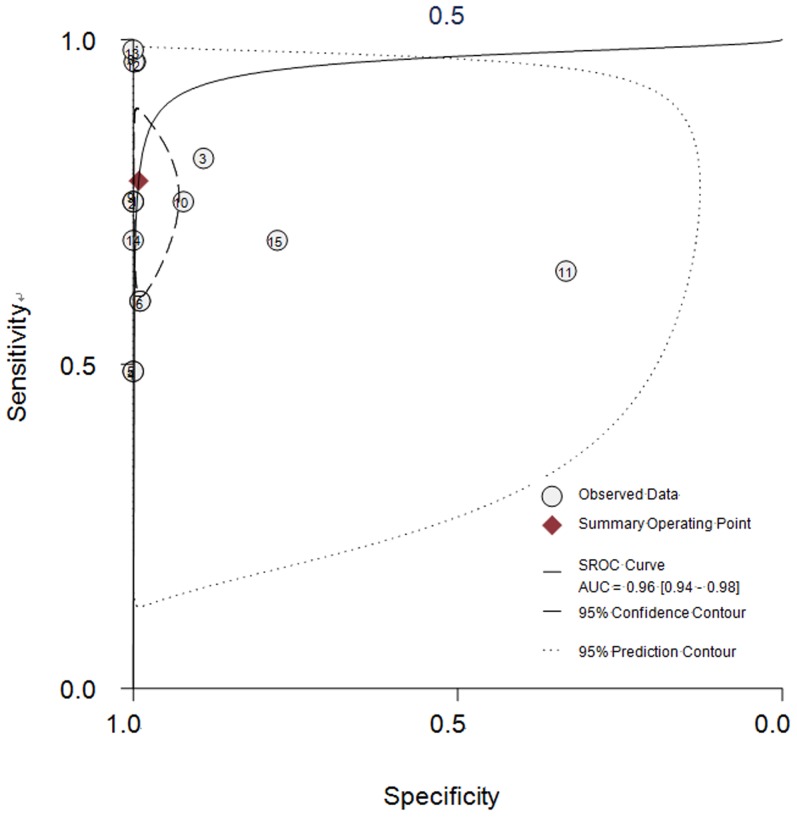
Summary receiver operating characteristic (SROC) graph with 95% confidence region and 95% prediction region for the diagnosis value of iMN by PLA2R-AB. Black square represents the summary estimate of sensitivity and specificity with the 95% confidence ellipse from the bivariate model. Numbers represent the reference numbers.

As shown in Figure 3, the summary sensitivity and specificity of all studies was 78% (95% CI: 66% to 87%) and 99% (95% CI: 96% to 100%), respectively. I^2^ was 93.65% for the summary sensitivity and 93.83% for the summary specificity, suggesting a high heterogeneity in the sample of studies. The area under the receiver operating characteristic curve was 0.96 (95% CI: 0.94 to 0.98) and the DOR was 437 (95% CI: 74 to 2592). The SROC graph with the 95% confidence region and the 95% prediction region are shown in Figure 4. The summary positive likelihood ratio was 96.1 (95% CI: 19.5 to 472.1) and the summary negative likelihood ratio was 0.22 (95% CI: 0.14 to 0.35).

A subgroup analysis was then performed to explore potential sources of heterogeneity among studies. As shown in Table 2, the diagnostic accuracy of PLA2R-AB testing was higher in Caucasian patients than in Asian patients. Similar findings were found in subgroups with the following characteristics: larger sample sizes, healthy controls, the use of an indirect immunofluorescence method, studies have test interval between two tests and studies that were not supported by government funding. In addition, a meta-regression analysis was conducted to identify any significant sources of heterogeneity. The results suggested that testing serum PLA2R-AB after the biopsy test was significantly associated with the accuracy of PLA2R-AB testing for the detection of iMN (*P* = 0.02).

**Table 2:** Subgroup analysis for accuracy of PLA2R-AB for MN detection.

Subgroup	N	Sensitivity	Specificity	DOR	AUC
Race					
Caucasian	10	0.80(0.62,0.91)	1.00(0.98,1.00)	1297	0.99(0.97–0.99)
Asian	5	0.72(0.67,0.77)	0.91(0.84,0.95)	26	0.91(0.88–0.93)
Sample size					
<100 subjects	6	0.82(0.66,0.91)	0.97(0.78,1.00)	147	0.94(0.92–0.96)
> = 100 subjects	9	0.76(0.59,0.88)	0.99(0.97,1.00)	465	0.97(0.96–0.99)
Control					
Patients	9	0.80(0.64,0.90)	0.98(0.89,1.00)	220	0.95(0.93–0.97)
Health	6	0.75(0.55,0.88)	1.00(0.98,1.00)	914	1.00(0.99–1.00)
Method					
Western blotting	9	0.68(0.62,0.73)	0.97(0.93,0.99)	73	0.84(0.80–0.87)
Imunofluorescence	4	0.74(0.37,0.93)	0.99(0.78,1.00)	451	0.98(0.96–0.99)
ELISA	1	-	-	-	-
Test interval					
No	8	0.68(0.62,0.73)	0.95(0.86,0.98)	39	0.78(0.74–0.81)
After biopsy test	7	0.86(0.65,0.96)	1.00(0.98,1.00)	8710	1.00(0.99–1.00)
Funding by					
Government foundation	9	0.70(0.56,0.81)	0.99(0.90,1.00)	177	0.89(0.86–0.92)
Corporation or no funding	6	0.88(0.76,0.95)	0.99(0.96,1.00)	843	0.99(0.98–1.00)

A sensitivity analysis was performed to evaluate the effect of each individual study on the pooled accuracy of PLA2R-AB testing. As shown in Table 3, a series of pooled sensitivity, specificity, DOR and area under curve (AUC) with 95% CIs were estimated after the removal of each study. The diagnostic accuracy of PLA2R-AB for iMN detection was relatively stable, with the exception of the study conducted by Dahnrich et al (2013).

**Table 3:** Sensitivity analyses for the accuracy of PLA2R-AB test on iMN detection.

Study omited	Sensitivity	Specificity	DOR	AUC
Beck	0.78(0.65,0.88)	0.99(0.95,1.00)	338(54.2114)	0.96(0.94–0.98)
Qin	0.78(0.65,0.87)	0.99(0.96,1.00)	431(71,2611)	0.97(0.95–0.98)
Hoxha2011	0.82(0.70,0.89)	0.99(0.94,1.00)	298(50,1778)	0.96(0.94–0.98)
Murtas	0.81(0.68,0.89)	0.99(0.95,1.00)	592(62,5612)	0.96(0.94–0.98)
Hoxha2012	0.75(0.64,0.84)	0.99(0.96,1.00)	326(58,1840)	0.94(0.92–0.96)
Svobodova	0.79(0.66,0.88)	0.99(0.97,1.00)	581(114,2951)	0.98(0.96–0.99)
Dahnrich	0.71(0.61,0.79)	0.99(0.93,1.00)	226(34,1522)	0.89(0.86–0.92)
Oh	0.80(0.66,0.89)	0.99(0.97,1.00)	601(89,4062)	0.97(0.96–0.98)
Zhou	0.79(0.65,0.88)	0.99(0.96,1.00)	567(75,4286)	0.97(0.95,0.98)

The funnel plot with a superimposed regression line is shown in Figure S1. The *P* value for the slope coefficient was less than 0.001, indicating significant asymmetry. This result suggests a potential publication bias among studies.

## Discussion

PLA2R is a major target antigen in autoimmune idiopathic membranous nephropathy [18]–[20]. Antibodies against PLA2R may serve as a new diagnostic biomarker for iMN detection. The methods for detecting PLA2R-AB, and the relationship between antibody concentration and its clinical manifestation, are not well known. Our meta-analysis suggests that the diagnostic accuracy of the serum PLA2R-AB test for iMN detection is modest, with a summary sensitivity of 78.0% and a specificity of 99%. Most of the studies included here have modest methodological quality. Moreover, the test time interval (whether or not biopsy was performed simultaneously) is a significant source of heterogeneity among studies.

To date, the etiology of iMN is not well understood. The diagnosis of iMN is still made by the exclusion of secondary causes, using the patient's medical history, physical examination, appropriate laboratory tests and renal pathological classification. Our meta-analysis suggested that PLA2R-AB may play a role in the development of iMN and might be a biomarker to help diagnose of iMN. However, the heterogeneity among studies suggested there are some potential factors that might have impact on the diagnostic accuracy of this test.

There are several possible explanations for why the testing interval is a significant source of the heterogeneity found in our meta-analysis. First, the various stages of disease may have been achieved during the testing interval. Second, the effect of immunosuppressive therapy may also be taken into account. If serum samples were collected long after the histological diagnosis, the patient may have entered an immunologically inactive stage by the time of serum collection, at which point the antibody disappears. As previously reported in Svobodova's study (2013), circulating anti-PLA2R was measured in 37 MGN patients several months after kidney biopsy and all patients had entered spontaneous or drug-induced remission. They found that the sensitivity of anti-PLA2R was 22% (8 of 37), compared with 64% that were positive at the initial diagnosis [18]. Therefore, we suggest serological testing should be performed at the time of initial diagnosis, rather than a period of time after renal biopsy. This would thus avoid the possible confound of therapeutic intervention and disease progression.

The detection efficiency of three methods Western blotting, immunofluorescence, and ELISA is controversial in studies examining the detection of serum PLA2R-AB levels [14]. According to our findings, it seems that the immunofluorescence method has higher diagnostic accuracy than Western blotting. However, both methods have their advantages and disadvantages. Most laboratories choose to use recombinant PLA2R1 as a substrate for immunofluorescence even though it is insufficient to assess antibody concentrations [14], [20], [21]. On the other hand, while Western blotting uses a monoclonal antibody to confirm the location of the PLA2R band, it has relatively high laboratory demands and the assessment of a large number of clinical patients can become complicated and cumbersome [11], [13]. Nevertheless, Debiec (2011) reported concordant resultsforPLA2R-AB in 42 iMN patients with both methods: Western blotting under non-reducing conditions and using glycoproteins extracted from normal human glomeruli; and immunofluorescence assay with HEK293 cells that were transfected with PLA2R1 cDNA [22], [23]. More recently, ELISA has also served as a promising method for the detection of PLA2R-AB. For example, Dahnrich (2013) reported that the ELISA test for PLA2R-ABhad 96.5% sensitivity and 100% specificity [20]. However, since there were not sufficiently available studies on ELISA test evaluation, we did not include the ELISA test in our meta-analysis.

PLA2R-AB was not found in all cases of iMN. This discrepancy could be partially due to the spontaneous remission of the disease or to the use of immunosuppressive therapy [5]. On the other hand, it is possible that iMN is not a uniform disease and might have different target antigens that have not yet been identified [24]. Our meta-analysis showed that the summary sensitivity of PLA2R-AB in all studies was only 78%. Svobodova (2013) suggested that the assessment of both circulating PLA2R antibodies and PLA2R antigen in biopsy specimens might be a better discriminator between primary and secondary MN than only assessing the levels of anti-PLA2R antibodies [18]. In some patients, MN can appear months or even years before a secondary cause is detected. Some patients who were negative for PLA2R might have been misclassified as idiopathic when they actually had a secondary form of MN [18]. This can only be examined by long-term, follow-up studies and further research on other antigens and the pathomechanism of iMN should be done in the future.

There are several limitations within this study that must be acknowledged. First, PLA2R-AB is a recently discovered biomarker, so few studies were available for our meta-analysis and our results might change as more work is done with PLA2R-AB. Second, the methodological quality of the included studies was not high. For example, the majority of studies did not report whether the serological examination results were obtained while blind to the kidney biopsy results. Such methodological limitations might have biased our final conclusions. Third, the potential publication bias among the selected studies indicates that the diagnostic value of PLA2R-AB on iMN detection may be overestimated, since studies with favorable results are more likely to be published.

In summary, the present meta-analysis suggests that there is modest diagnostic value in serum PLA2R-AB testing for the detection of iMN. Considering our limitations and the heterogeneity among our chosen studies, large and well-designed prospective studies will be needed to determine the future diagnostic value of serum PLA2R-ABtesting.

## Supporting Information

Figure S1 **Deeks' Funnel Plot Asymmetry Test.** Funnel plot of the natural logarithm of the diagnostic odds ratio(lnDOR) against the inverse of the square root of the effective sample size (1/ESS1/2) of included studies.
(TIF)

Table S1 **The characters detail of included studies.**
(DOCX)

Table S2 **The QUADAS form for included studies.**
(DOCX)

Checklist S1 PRISMA checklist. Preferred Reporting Items for Systematic Reviews and Meta-Analyses.
(DOC)
