# Supplementary figures and images for: The Diagnosis Accuracy of PLA2R-AB in the Diagnosis of Idiopathic Membranous Nephropathy: A Meta-Analysis
Source: PLoS One. 2014 Aug 19;9(8):e104936. doi: 10.1371/journal.pone.0104936 (PMC4138154; doi:10.1371/journal.pone.0104936)

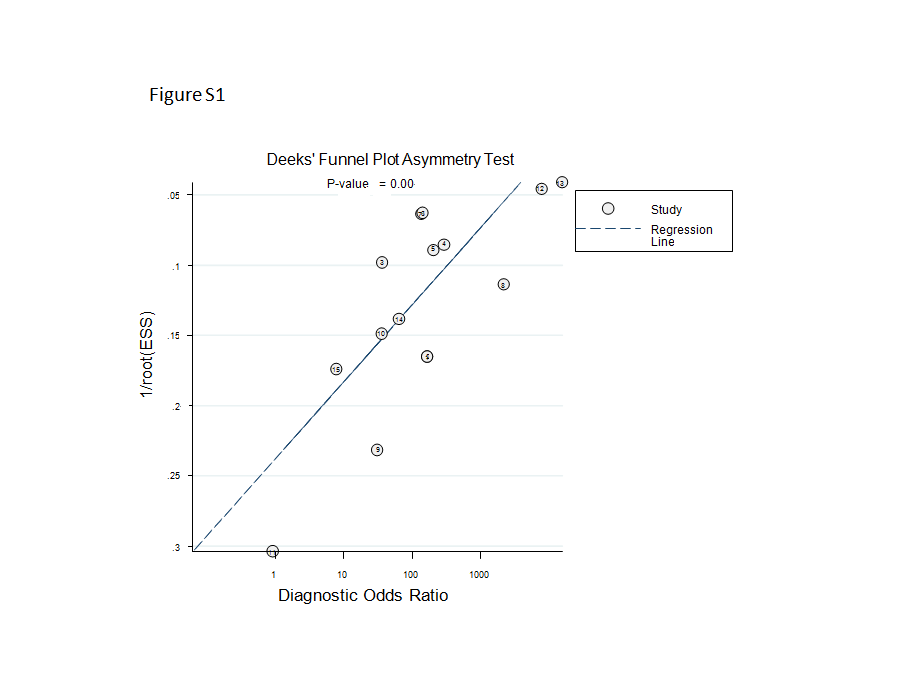

Supplement: Figure S1 — Deeks' Funnel Plot Asymmetry Test. Funnel plot of the natural logarithm of the diagnostic odds ratio(lnDOR) against the inverse of the square root of the effective sample size (1/ESS1/2) of included studies. (TIF) [file pone.0104936.s001.tif]
