# Supplementary material for: The Diagnosis Accuracy of PLA2R-AB in the Diagnosis of Idiopathic Membranous Nephropathy: A Meta-Analysis
Source: PLoS One. 2014 Aug 19;9(8):e104936. doi: 10.1371/journal.pone.0104936 (PMC4138154; doi:10.1371/journal.pone.0104936)
Supplement: Table S1 — The characters detail of included studies. (DOCX) [file pone.0104936.s002.docx]

**Table S1 The characters detail of included studies.**

| Year | Author | Country | Race | Sex(MN) | Sex(control) | Average Age(years) | Average Age(years) | Study design |
| --- | --- | --- | --- | --- | --- | --- | --- | --- |
| 2009 | Beck | USA | Caucasian | MN(+):M/F=6/3  MN(-):M/F=2/1 | Secondary MN:M/F=1/7  Disease controls: M/F=10/12  Normal controls: M/F=15/15 | MN(+):51.6  MN(-):47.7 | Secondary MN:39.9  disease controls:48.5  Normal controls:51.0 | retrospective |
| 2011 | Weisong | China | Asia | MN(+):M/F=40/9  MN(-):M/F=4/7 | Lupus-MN:M/F=4/16  HBV-MN:M/F=13/3  tumer MN:M/F=9/1 | MN(+):49.0  MN(-):39.2 | Lupus-MN:32.5  HBV-MN:32.2  tumer MN:53.6 | prospective |
| 2011 | Elion Hoxha | Germany | Caucasian | NA | NA | MN: NA | Secondary MN:49.6  non-membranous glomerulonephritis:48.2 | retrospective |
| 2012 | Corrado | Italian | Caucasian | MN：M/F=121/65 | FSGS：M/F=19/13  IgAN：M/F=38/22  Normal：M/F=56/40 | MN:59±16 | FSGS:18±3  IgAN:40±4  Normal:49±10 | retrospective |
| 2012 | Elion Hoxha | Germany | Caucasian | MN: M/F=49/12 | Secondary MN+ No known cause of secondary MN: M/F=15/12 | MN：56.5±15.8 | Secondary MN+ No known cause of secondary MN:57.7±15.0 | prospective |
| 2013 | Barbora | Czech | Caucasian | MN(+):M/F=31/14  MN(-):M/F=16/4 | NA | MN(+):52.6±13.6  MN(-):55.0±14.3 | NA | retrospective |
| 2013 | Dahnrich | West Europe | Caucasian | MN:M/F=133/67 | Secondary MN:M/F=15/12  Other renal disease: M/F=150/80  Other autoimmune disease: M/F=54/262  Healthy individuals: M/F=179/112 | MN:54(16~86) | Secondary MN:57(25~79)  other renal disease:43.3  other autoimmune disease:55.1  Healthy individuals:38(18~68) | retrospective |
| 2013 | Yun | Korea | Asia | MN(+):M/F=40/29  MN(-):M/F=13/18 | NA | MN(+):55.1±12.7  MN(-):53.8±16.5 | NA | prospective |
| 2012 | Guangyu | China | Asia | NA | NA | NA | NA | prospective |
