# Supplementary material for: The Diagnosis Accuracy of PLA2R-AB in the Diagnosis of Idiopathic Membranous Nephropathy: A Meta-Analysis
Source: PLoS One. 2014 Aug 19;9(8):e104936. doi: 10.1371/journal.pone.0104936 (PMC4138154; doi:10.1371/journal.pone.0104936)
Supplement: Table S2 — The QUADAS form for included studies. (DOCX) [file pone.0104936.s003.docx]

**Table S2 The QUADAS form for included studies.**

| QUADAS items | Variables | Summary  Score | Percentage(%) | 2009 | 2011 | 2011 | 2012 | 2012 | 2013 | 2013 | 2013 | 2012 |
| --- | --- | --- | --- | --- | --- | --- | --- | --- | --- | --- | --- | --- |
|  |  |  |  | Beck | Qin | Hoxha | Murtas | Hoxha | Svobodoa | Dähnrich | Oh | Zhou |
| 1. Was the spectrum of patients representative of the patients who will receive the test in practice? | Q1 | 9 | 100 | 1 | 1 | 1 | 1 | 1 | 1 | 1 | 1 | 1 |
| 2. Were selection criteria clearly described? | Q2 | 9 | 100 | 1 | 1 | 1 | 1 | 1 | 1 | 1 | 1 | 1 |
| 3. Is the reference standard likely to correctly classify the target condition? | Q3 | 9 | 100 | 1 | 1 | 1 | 1 | 1 | 1 | 1 | 1 | 1 |
| 4. Is the time period between reference standard and index test short enough to be reasonably sure that the target condition did not change between the two tests? | Q4 | 5 | 56 | 0 | 1 | 0 | 1 | 0 | 1 | 0 | 1 | 1 |
| 5. Did the whole sample or a random selection of the sample, receive verification using a reference standard of diagnosis? | Q5 | 9 | 100 | 1 | 1 | 1 | 1 | 1 | 1 | 1 | 1 | 1 |
| 6. Did patients receive the same reference standard regardless of the index test result? | Q6 | 9 | 100 | 1 | 1 | 1 | 1 | 1 | 1 | 1 | 1 | 1 |
| 7. Was the reference standard independent of the index test (i.e. the index test did not form part of the reference standard)? | Q7 | 9 | 100 | 1 | 1 | 1 | 1 | 1 | 1 | 1 | 1 | 1 |
| 8. Was the execution of the index test described in sufficient detail to permit replication of the test? | Q8 | 7 | 78 | 1 | 1 | 1 | 0 | 1 | 0 | 1 | 1 | 1 |
| 9. Was the execution of the reference standard described in sufficient detail to permit its replication? | Q9 | 9 | 100 | 1 | 1 | 1 | 1 | 1 | 1 | 1 | 1 | 1 |
| 10. Were the index test results interpreted without knowledge of the results of the reference standard? | Q10 | 1 | 11 | 0 | 0 | 0 | 0 | 0 | 1 | 0 | 0 | 0 |
| 11. Were the reference standard results interpreted without knowledge of the results of the index test? | Q11 | 9 | 100 | 1 | 1 | 1 | 1 | 1 | 1 | 1 | 1 | 1 |
| 12. Were the same clinical data available when test results were interpreted as would be available when the test is used in practice? | Q12 | 8 | 89 | 1 | 1 | 1 | 1 | 1 | 0 | 1 | 1 | 1 |
| 13. Were uninterpretable/ intermediate test results reported? | Q13 | 9 | 100 | 1 | 1 | 1 | 1 | 1 | 1 | 1 | 1 | 1 |
| 14. Were withdrawals from the study explained? | Q14 | 9 | 100 | 1 | 1 | 1 | 1 | 1 | 1 | 1 | 1 | 1 |
| Summary score |  |  |  | 12 | 13 | 12 | 12 | 12 | 12 | 12 | 13 | 13 |
